# The physical map of wheat chromosome 5DS revealed gene duplications and small rearrangements

**DOI:** 10.1186/s12864-015-1641-y

**Published:** 2015-06-13

**Authors:** Bala Ani Akpinar, Federica Magni, Meral Yuce, Stuart J. Lucas, Hana Šimková, Jan Šafář, Sonia Vautrin, Hélène Bergès, Federica Cattonaro, Jaroslav Doležel, Hikmet Budak

**Affiliations:** Sabanci University Nanotechnology Research and Application Centre (SUNUM), Sabanci University, Universite Cad. Orta Mah. No: 27, Tuzla, 34956 Istanbul Turkey; Instituto di Genomica Applicata, Via J.Linussio 51, Udine, 33100 Italy; Centre of the Region Haná for Biotechnological and Agricultural Research, Institute of Experimental Botany, CZ-78371 Olomouc, Czech Republic; Centre Nationales Ressources Génomiques Végétales, INRA UPR 1258, 24 Chemin de Borde Rouge - Auzeville 31326, Castanet-Tolosan, France; Molecular Biology, Genetics and Bioengineering Program, Sabanci University, 34956 Istanbul, Turkey

**Keywords:** *Triticum aestivum*, 5DS, Hexaploid wheat, Physical mapping, Gene space, Grass evolution

## Abstract

**Background:**

The substantially large bread wheat genome, organized into highly similar three sub-genomes, renders genomic research challenging. The construction of BAC-based physical maps of individual chromosomes reduces the complexity of this allohexaploid genome, enables elucidation of gene space and evolutionary relationships, provides tools for map-based cloning, and serves as a framework for reference sequencing efforts. In this study, we constructed the first comprehensive physical map of wheat chromosome arm 5DS, thereby exploring its gene space organization and evolution.

**Results:**

The physical map of 5DS was comprised of 164 contigs, of which 45 were organized into 21 supercontigs, covering 176 Mb with an N50 value of 2,173 kb. Fifty-eight of the contigs were larger than 1 Mb, with the largest contig spanning 6,649 kb. A total of 1,864 molecular markers were assigned to the map at a density of 10.5 markers/Mb, anchoring 100 of the 120 contigs (>5 clones) that constitute ~95 % of the cumulative length of the map. Ordering of 80 contigs along the deletion bins of chromosome arm 5DS revealed small-scale breaks in syntenic blocks. Analysis of the gene space of 5DS suggested an increasing gradient of genes organized in islands towards the telomere, with the highest gene density of 5.17 genes/Mb in the 0.67-0.78 deletion bin, 1.4 to 1.6 times that of all other bins.

**Conclusions:**

Here, we provide a chromosome-specific view into the organization and evolution of the D genome of bread wheat, in comparison to one of its ancestors, revealing recent genome rearrangements. The high-quality physical map constructed in this study paves the way for the assembly of a reference sequence, from which breeding efforts will greatly benefit.

**Electronic supplementary material:**

The online version of this article (doi:10.1186/s12864-015-1641-y) contains supplementary material, which is available to authorized users.

## Background

Cereals are the primary components of human nutrition worldwide. Among the cereals, wheat ranks the third in global production after rice and maize, comprising over 650 million tons of approximately 2.3 billion tons of cereals produced annually. Of the cereals allocated for human consumption, wheat and rice are the main contributors (FAO Statistical Yearbook, 2013). The relatively small (389 Mb) genome of rice has been fully sequenced and annotated [1] and a draft genome sequence is available for the 2.3 Gb maize genome [2]. Recently, the draft genome sequences of two progenitors of bread wheat, *Triticum urartu* and *Aegilops tauschii* genomes, have been published [3, 4]. The third progenitor of wheat remains unknown, and the diploid grass *Aegilops speltoides* with its S genome is the closest identified relative of the B genome of wheat [5]. Although the reference sequence of the entire bread wheat genome is far from complete, a chromosome-based draft sequence has just been published [6].

Bread wheat (*Triticum aestivum* L.) originated from a spontaneous hybridization between the cultivated tetraploid wheat *Triticum turgidum* L. (2n = 4x = 28, AABB genome) and the wild diploid grass *Aegilops tauschii* Coss. (2n = 2x = 14, DD genome), followed by genome duplication, forming its hexaploid genome (2n = 6x = 42, AABBDD genome) [7, 8]. Accordingly, the allohexaploid wheat genome is not only huge (~17 Gb) in size; but also complex due to the A, B and D sub-genomes, which contain numerous paralogous and homeologous loci. A further complication to whole-genome sequencing efforts is the repeat content, which is estimated to represent over 80 % of the genome [9, 10]. Despite the advent of next-generation sequencing technologies, the above mentioned attributes of the wheat genome have rendered the assembly of genomic sequences extremely difficult. A break-through in wheat genomics has been achieved in the recent years, as advances in chromosome flow-sorting techniques have enabled genomics studies based on isolated chromosomes [11, 12]. The so-called “chromosome-by-chromosome” approach proposed by the International Wheat Genome Sequencing Consortium (IWGSC) has been validated on the largest chromosome of the wheat genome, the ~1 Gb 3B chromosome, ultimately sequenced to the reference quality [13, 14]. Following chromosome 3B, five additional physical maps have been constructed for the short and long arms of chromosome 1A and 1B, and finally chromosome 6A [15–19].

In the absence of a finished quality genome sequence, insights into wheat genome structure and function have been accumulating through survey sequencing of individual chromosomes or chromosome-specific Bacterial Artificial Chromosome (BAC) libraries. So far, survey sequences for wheat chromosomes 4A, 5A, 5D, 6B, 7BS and 7DS have been published [20–25]. In particular, comparative analyses of the 5D chromosome with its counterpart in the wild progenitor, *Ae. tauschii*, provided valuable insights into the wheat genome evolution [25, 26]. Additionally, BAC-end sequences (BES) of wheat chromosomes 3B, 1AL and 3AS [27–29] and sequencing of selected BAC clones covering different regions of chromosome 3B [10] have been informative on the composition and organization of the wheat genome. Very recently, chromosome-specific shotgun sequences of all bread wheat chromosomes have become available [6], along with the first reference sequence of the chromosome 3B [14]. The sequence information obtained from these studies has enabled exploration of both coding and non-coding regions. Recently, the entire wheat genome has been sequenced to 5x coverage, enabling in-depth exploration of gene-derived sequences [30]. However, establishing a ‘finished quality’ genome sequence of the bread wheat genome with accurate positioning of genes along the chromosomes remains elusive.

Despite the vast knowledge gathered so far, the utilization of the wheat genome to its full extent requires the completion of a reference genome sequence. Due to the hexaploidy and highly repetitive nature of the genome, integrated physical and genetic maps are essential to the assembly of the high-throughput sequence data into a finished quality reference sequence. Validation of the feasibility of physical map construction from chromosome-specific BAC libraries (http://olomouc.ueb.cas.cz/dna-libraries/cereals) has set the pace for this ultimate goal; currently, physical mapping of other chromosome-specific BAC libraries are underway (www.wheatgenome.org/Projects).

While the first physical maps of the wheat genome relied on the FingerPrintedContig (FPC) software [31] for the assembly of the BAC clones, the recently developed Linear Topology Contig (LTC) software has been suggested to build longer and fewer contigs, thus improving the map quality [32]. Indeed, in our previous study on 1AL chromosome, LTC generated an assembly of 583 contigs with the N50 contig size, which is considered as a quality measure of the physical map, of 1,166 kb, whereas FPC generated assembly consisted of 1,180 contigs with an N50 contig size of 460 kb [15]. Accordingly, the more recently published 1BL physical map constructed by the FPC software was integrated with the LTC-generated assembly [16]. In this study, we present the physical map of chromosome 5DS, entirely constructed by the LTC software. As the first physical map of the D-genome, the physical map of the 5DS chromosome of bread wheat expands the opportunities to study wheat genome evolution and domestication, and develop molecular markers and tools for gene cloning and genomics assisted breeding.

## Results and Discussion

### Construction of the 5DS physical map

Flow cytometric analysis of DAPI (4',6-diamidino-2-phenylindole)-stained mitotic metaphase chromosomes isolated from double ditelosomic line 5D of cv. Chinese Spring [33] resulted in flow karyotypes in which peaks representing the short and long arms of 5D were clearly discriminated, enabling their sorting (Additional file 1). The analysis of flow-sorted fractions by Fluorescence in situ Hybridization (FISH) indicated 88 % purity, with the sorted fractions contaminated by a random mix of fragments from various other chromosomes and chromatids. A 5DS-specific BAC library designated TaaCsp5DShA was constructed from DNA of 8,120,000 sorted 5DS arms. The ordered library comprised 36,864 BAC clones with average insert size of 137 kb, representing 17x coverage of the 258 Mb-long chromosome arm [34]. Of this library, 26,112 BAC clones with an average insert size of 143 kb, giving approximately 12.5x coverage of the arm, were fingerprinted using SNaPshot™ High-Information Content Fingerprinting (HICF) procedure [35]. Good-quality fingerprints were obtained for 21,656 clones (82 % of the clones that were fingerprinted) and used to construct the physical map of 5DS.

A robust tool for constructing physical maps from fingerprinted BAC libraries has been the FingerPrintedContig (FPC) software, which was also adopted by the International Wheat Genome Sequencing Consortium (IWGSC) for physical mapping of wheat chromosomes. As an alternative to FPC, Linear Topological Contig (LTC) software has been introduced recently and reported to build fewer and longer contigs [32]. LTC also enables evaluation of the contig topology; disruptions of the linear chromosome structure, indicating problematic clone overlaps that can then be corrected or avoided. Initially, both software programs, namely FPC and LTC, were separately implemented in the physical map construction. The resulting preliminary maps were compared to determine the most reliable one to work on further. Although the estimated coverage of the chromosome arm by FPC-constructed map was greater than LTC-constructed map (78 % versus 68 %), the number of contigs in LTC-constructed map was considerably less than that of FPC, while the N50 contig size was almost twice as large (Table 1). Additionally, the largest contig size with the LTC-map was markedly higher than that of FPC-map (6,649 kb versus 4,053 kb). Consequently, LTC-constructed preliminary map was used for further analyses.

**Table 1:** Comparison of FPC and LTC assemblies of 5DS physical map

	FPC assembly	LTC assembly
Total no. of clones	21656	21656
Number of contigs (>5 clones)	350	120
MTP clones	1894	2155^a^
Assembly length	202728	176838
Average contig size	579	1078
N50	1141 kb	2173 kb
L50	53	27
Contigs > 1 Mb	63	58

^a^Picked by FPC software

The minimum tiling path (MTP) originally picked by LTC in the LTC-constructed map was found to contain several buried clones. Thus, MTP clones were re-selected using FPC on the LTC-constructed map, which resulted in the inclusion of longer clones in the MTP. The MTP clone overlaps were also tested using LTC. Since LTC software requires a significance cut-off of 10^−15^ to build overlapping clones, any clone overlaps significant at a less stringent cut-off can be considered as unreliable. For contigs with accurate ordering of clones, such low quality overlaps may result from missing bands in the fingerprints, which was the case for the contigs in the LTC-map that were reported to contain unreliable clone overlaps. Therefore, all clone overlaps that were significant only at a cut-off of 10^−14^ or above were reinforced by adding 210 additional clones that covered the same overlap region, to avoid any gaps at the sequence level. Finally, 163 more clones, labeled as Q-clones by LTC, were also included in the MTP. These clones appeared to cluster into 2 or more contigs, and so could either be chimaeric clones, or genuine bridge clones between different contigs in areas of low coverage. Therefore, although they are considered questionable clones, if supported by molecular marker data they may be valuable for merging contigs. After these additions, the FPC-picked and manually edited MTP of the LTC-constructed preliminary map of 5DS contained a total of 2,528 clones. This preliminary map will be referred as 5DS preliminary map from this point on. This 5DS physical map is available at https://urgi.versailles.inra.fr/gb2/gbrowse/wheat_phys_pub/.

### Assessment of the 5DS preliminary map

The LTC-constructed 5DS preliminary map was estimated to cover over 68 % of the entire 258 Mb-long chromosome arm with an assembly length of 176 Mb. This assembly was composed of 120 contigs containing at least 6 clones and 44 short contigs containing 5 or less clones, giving rise to a total of 164 contigs overall. The N50 value of the preliminary map was 2,173 kb, indicating that 50 % of the assembly was covered with contigs longer than this value. The number of such contigs (L50 value) was 27. When short contigs were excluded, N50 was raised to 2,226 kb, for a total number of 120 contigs with an assembly length of 168 Mb (covering 65 % of 5DS). As a quality measure, the N50 value of 5DS preliminary map (2,173 kb including short contigs) compared to previous LTC-constructed maps of 1AL (1,166 kb), 1BL (961 kb), 1AS (798 kb), 1BS (2,430 kb), 6AS (1106 kb) and 6AL (921 kb), indicates a high quality map for this chromosome arm [15-19].

**Fig. 1:**
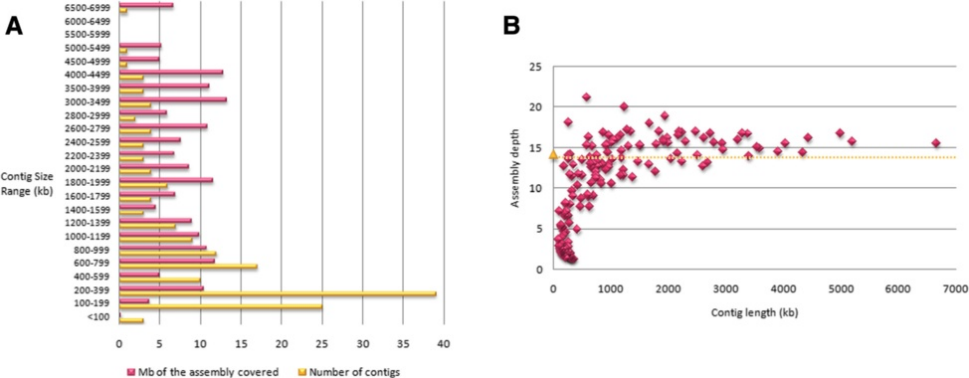
Contig length and depth of 5DS preliminary map. **a** Distribution of contig lengths across different size ranges. **b** Depth of assembly coverage by contig length

The average contig size of the 5DS preliminary map was 1,078 kb, with the longest contig size being 6,649 kb, comparable to the LTC-constructed physical maps of 1AL, 1BL and 1BS which ranged between 5.8 Mb to over 7 Mb [15, 16, 18]. A total of 58 of the 164 contigs were longer than 1 Mb. The contig sizes were distributed in size ranges of 100 kb for contigs smaller than 200 kb, 500 kb for contigs larger than 3,000 kb, and 200 kb for the rest of the contigs; then, plotted against the number of contigs and megabases of the assembly covered by the contigs in the respective size range (Fig. 1a). Although many contigs fall in the 100 – 400 kb range, the contribution of the ranges to the overall assembly length was observed to be roughly uniform across all size ranges. Additionally, the assembly depth of the contigs, calculated contig length (number of clones multiplied by the average insert size of 143 kb) divided by the actual length of the contig, were plotted against the length of contigs (Fig. 1b). As shown in Fig. 1b, though many small contigs were clustered around a depth of 1 – 5x, large contigs exhibited higher depths around 12 – 20x. The average assembly depth estimated by calculated assembly length divided by actual assembly length was 14x (indicated by the yellow dashed line), around which large contigs were clustered. 56 of the 164 contigs had 14x or higher coverage, all of which containing 26 or more clones. The preliminary map details of the 5DS are given in Additional file 2.

### Marker design and MTP screening

Recently published survey sequences of the 5DS chromosome from *T. aestivum* cv. Chinese Spring [25] were utilized to design 16,727 Insertion Site-Based Polymorphism (ISBP) and 75 Simple Sequence Repeat (SSR) markers to aid in contig anchoring and ordering. A total of 30 ISBP markers were physically anchored to specific clones of the MTP, thereby verifying these markers. The large number of these ISBP and SSR markers newly designed for 5DS presents a rich marker source which can be utilized in further studies (Additional file 3). In addition to these markers, to refine the 5DS preliminary map, the MTP clones were screened by a variety of molecular markers. Initially, a total of 23 SSR markers [6 BARC (the acronym for the USDA-ARS Beltsville Agricultural Research Center), 9 CFD, 3 WMC (Wheat Microsatellite Consortium), 4 WMS/GWM (Gatersleben Wheat Microsatellite) and 1 GPW), 13 COS (Conserved Orthologous Set) markers, 10 EST (Expressed Sequence Tag) markers and 2 gene-based markers (Pina-D1, Pinb-D1) that were genetically mapped to chromosome 5DS were used to screen the MTP pools. Of these, 13 SSR markers (56 %), 12 COS markers (92 %) and 2 gene-based markers (100 %) could be assigned to specific contigs, while of the 15 EST markers only 1 could be assigned to a specific contig. The low rate of anchoring EST markers to contigs was concluded to result from intronic sequences which are present on MTP clones but not on EST markers. Therefore, the sequences of genetically mapped EST markers were blasted against 1.34x coverage genomic survey sequences of 5DS [25] to define intron-exon boundaries; new primer pairs were then designed to amplify sequences from within a single or two closely adjacent exons. A total of 50 such EST markers were designed and tested on MTP pools and 43 of these (86 %) could be anchored to specific contigs. Interestingly, two EST markers, namely BF483719 and CD882766 that could not be anchored previously, were anchored to contigs 115 and 134, respectively, using the approach mentioned above. For the remaining 12 EST markers that could not be assigned to any contigs previously, the blast approach did not yield favorable sequences to be amplified. The relatively low success rate of the SSR markers could be due to sequence divergence among the wheat lines from which the SSRs were designed and tested. On the other hand, COS markers, designed from conserved genic sequences, and EST markers, targeting the expressed portion of the genome that is well conserved, could be anchored to 5DS contigs relatively easily, as expected. Overall, 48 of a total of 164 contigs of the 5DS preliminary map were physically anchored by at least 1 molecular marker via Polymerase Chain Reaction (PCR) (Additional file 4). At the upper extreme, two of these contigs were anchored by 6 molecular markers each.

Recently, a customized microarray hybridization approach was successfully applied to assign large numbers of markers to chromosome-specific BAC pools [15, 36]. Thus, a custom array was designed to include probes from gene, SSR, COS, Single Nucleotide Polymorphism (SNP) and EST markers that have been genetically mapped to 5DS, as well as probes from ISBP markers or conserved genes generated from 1.34x survey sequences of 5DS [25]. The hybridization of this 5DS-specific array to 5DS MTP pools enabled putative assignment of 1,767 unique gene or marker associated sequences to 3,066 MTP clones of the 5DS physical map at high stringency. Probes from 3 syntenic genes and 2 ISBPs yielded ambiguous assignments, and, thus, were discarded, leaving the total number of unique gene or marker associated sequences assigned to MTP clones as 1,762 (Additional file 4). In total, 25 % of SSR (5 out of 20), 23 % of COS (3 out of 13), 12 % of EST (15 out of 122), 15 % of SNP (17 of the 112), 18 % of conserved reads (1,306 out of 6,996), and 8 % of ISBP (416 out of 5,120) markers were putatively assigned to specific 5DS MTP clones. Previous studies utilized the wheat NimbleGen 40 k UniGene microarray [37] to putatively assign 1,122 and 1,615 UniGenes to 1AL [15] and 1BL [16] physical maps, respectively; the same approach yielded the assignment of 3,878 and 647 UniGenes to 1BS [18] and 1AS [17], respectively, as the two extremes. These differences in the number of UniGenes assigned to different chromosome arms may be due to differences in the size and structure of the respective chromosome arm; however, stringency levels applied may also account for the differences. Though the number of markers anchored to 5DS contigs through microarray appears relatively low compared to the 1BS physical map, of the 18 markers anchored by both PCR and microarray approaches, 17 were in complete agreement, suggesting that the high stringency applied minimized the false negatives, possibly at the cost of some true positives. While previous studies generally used the wheat NimbleGen 40 k UniGene microarray designed from the entire wheat genome [37], a 17 k ISBP NimbleGen array was designed specifically for 1BL, resulting in the anchoring of 3,912 ISBPs to the physical map [16] in a similar way to our custom array containing probes from a variety of sequence sources. These probe sequences could also be used to design new markers to physically anchor their assigned contigs in future studies.

**Fig. 2:**
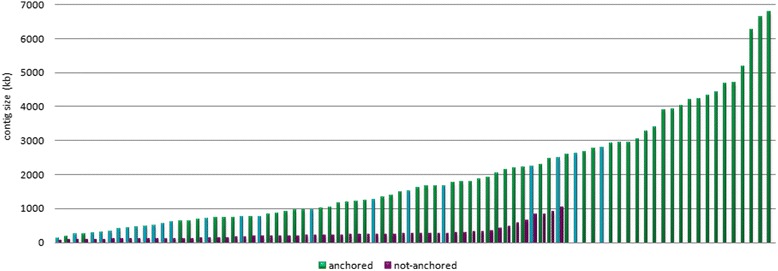
Contig sizes of the anchored and not anchored contigs. Green and blue bars indicate anchored contigs, where green bars correspond to anchored contigs that are also mapped to specific locations along the chromosome arm. Purple bars denote contigs that are not anchored by any molecular markers or hybridization probes

Through the above mentioned approaches, a total of 1,865 molecular markers were confidently anchored to 105 of the 164 contigs of the 5DS physical map, yielding a marker density of ~10.5 markers per Mb. This marker density was close to the marker density of the final physical map of 1BL (11 markers/Mb) [16] and exceeded that of 1BS (10.1 markers/Mb) [18], indicating a highly saturated map that should be of great utility in map-based cloning or marker-assisted genomics studies. Anchored contigs covered approximately 161 Mb of the total assembly length of 176 Mb (~91 %) of the physical map, indicating that the contigs remaining unanchored were mostly short contigs of little informative value (Fig. 2, purple bars). This conclusion is further corroborated when short contigs are excluded; 100 of the 120 contigs anchored by molecular markers cumulatively cover ~95 % of the total assembly (160 Mb of the 168 Mb).

### Ordering 5DS contigs along the chromosome arm

Contigs of the 5DS preliminary physical map were ordered along the chromosome arm using genetically mapped marker data and syntenic relationships. For genetically mapped SSR, EST and COS markers, previously published mapping data was used; whereas deletion-bin mapping was performed for ISBP markers physically anchored to 5DS map. Recently, the physical map of the entire *Aegilops tauschii* genome, which exhibits extensive similarities with the bread wheat D-genome due to their relatively recent hybridization, has been published [35]. Consequently, 5DS contigs allocated to chromosome deletion bins by genetically mapped markers were ordered within each bin based on the order of their ortholog sequences on *Ae. tauschii* 5D chromosome. For contigs that did not yield a significant homology to *Ae. tauschii* sequences, the order along the genome zipper of 5DS constructed from the syntenic relationships with the *Brachypodium* chromosome 4 by our group was retained [25]. Contigs without significant matches to *Ae. tauschii* 5D map or 5DS genome zipper could only be allocated to relatively large deletion bins and the orders of these contigs within the bins remained unclear.

**Fig. 3:**
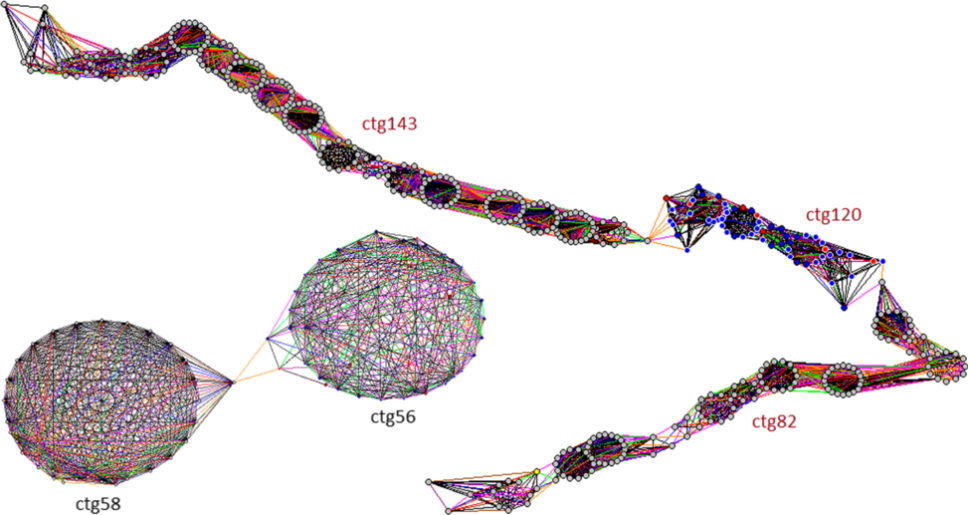
Representative supercontigs. The net of clone overlaps for supercontig[CTG58-CTG56] which is made up of 2 contigs, and supercontig[CTG143-CTG120-CTG82] which incorporates 3 contigs. Vertices indicate individual BAC clones and edges with different coloring indicate clone overlaps with different levels of significance. The net of clone overlaps are visualized by LTC

To further aid in the ordering, contig ends were manually checked for contig elongation into supercontigs. 21 supercontigs were constructed by manually elongating the ends of a total of 45 contigs, including those with less than six clones. The network of clone overlaps for two representative supercontigs are given in Fig. 3. The complete list of all supercontigs is given in Table 2, and the details of all 21 supercontigs are found in Additional file 5, with connecting clones indicated. Excluding short contigs, the total number of supercontigs remained as 19, comprising of 41 contigs of at least six clones.

**Table 2:** Supercontigs built from 5DS contigs

Supercontig No.	contigs	# of clones	total # of clones	status
SC1	[CTG98-CTG54-CTG68]	86, 21, 583	690	mapped
SC2	[CTG56-CTG58]	38, 33	71	mapped
SC3	[CTG57-CTG162]	74, 101	175	anchored
SC4	[CTG66-CTG122]	34, 135	169	mapped
SC5	[CTG70-CTG145-CTG146]	231, 14, 104	349	mapped
SC6	[CTG71-CTG100]	26, 318	344	mapped
SC7	[CTG74-CTG109]	9, 9	18	anchored
SC8	[CTG77-CTG127]	138, 393	531	mapped
SC9	[CTG79-CTG80]	11, 3	14	anchored
SC10	[CTG143-CTG120-CTG82]	251, 61, 136	448	mapped
SC11	[CTG88-CTG90]	38, 96	134	mapped
SC12	[CTG92-CTG64]	99, 2	101	Not anchored
SC13	[CTG111-CTG112]	211, 62	273	mapped
SC14	[CTG158-CTG118]	224, 70	294	mapped
SC15	[CTG144-CTG121]	372, 113	485	mapped
SC16	[CTG136-CTG148]	218, 124	342	anchored
SC17	[CTG140-CTG149]	217, 256	473	mapped
SC18	[CTG159-CTG150]	76, 62	138	mapped
SC19	[CTG131-CTG151]	48, 16	64	mapped
SC20	[CTG157-CTG156]	358, 373	731	mapped
SC21	[CTG105-CTG108]	28, 26	54	anchored

*SC*: Supercontig; *mapped*: anchored by 1 or more markers and mapped to a deletion bin on 5DS; *anchored*: anchored by 1 or more markers; *notanchored*: not anchored by any molecular markers

**Fig. 4:**
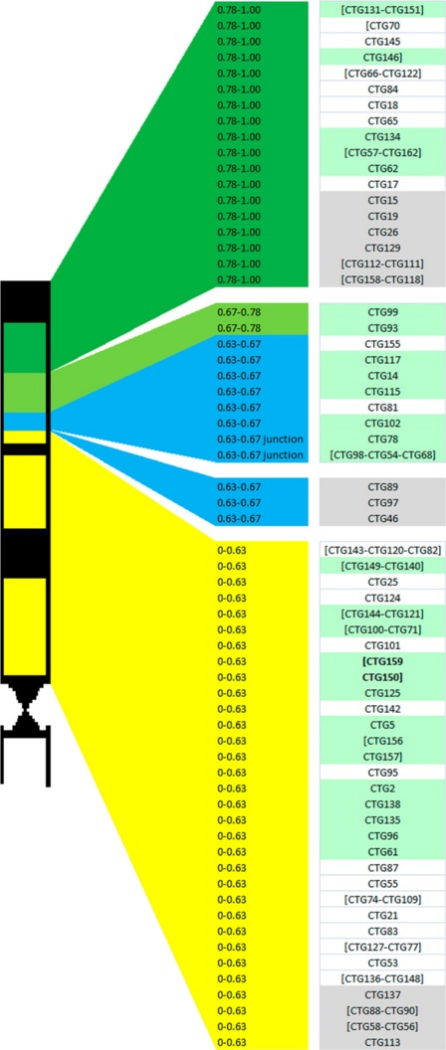
Deletion bin map of 5DS contigs, aided by genetically mapped molecular markers/probes and genome conservation. Different deletion bins are indicated by different coloring. Chromosome idiogram is taken from: https://www.ksu.edu/wgrc/Germplasm/Deletions/group5.html

Construction of the supercontigs and the genetically mapped molecular markers along with synteny enabled allocation of 80 contigs (39 contigs in 18 supercontigs and 41 contigs) and 79 (39 contigs in 18 supercontigs and 40 contigs) of the 105 and 100 anchored contigs of the 5DS physical map with or without short contigs, respectively, into 4 cytogenetically defined deletion bins (https://www.ksu.edu/wgrc/Germplasm/Deletions/group5.html, Additional file 6). While the number of anchored contigs to the genetic map of 5DS was considerably higher than that of 1AL physical map [15], more recent physical maps of 1BS, 1BL, and 6A reported similar percentages of contigs integrated with the genetic maps, as 77.4 %, 74 %, and 79 %, respectively [16, 18, 19]. Among these contigs, 63 were ordered within the deletion bins utilizing primarily the shared extensive homology between *Ae. tauschii* and *T. aestivum* and, secondarily, syntenic relationships with the model grass *Brachypodium distachyon* (Fig. 4). While the most distal bin, 0.78-1.00, contained 23 contigs, the most proximal 0–0.63 bin contained 42 contigs. Interestingly, 13 contigs were mapped to the relatively narrow 0.63-0.67 cytogenetic interval, while only 2 could be assigned to the 0.67-0.78 bin. Additionally, contig CTG93 assigned to the 0.67-0.78 deletion bin by related genomes, was also anchored by CFD81 SSR marker which is mapped to the most proximal 0–0.63 bin [38]. Although contig CTG78 and supercontig [CTG98-CTG54-CTG68] were assigned to deletion interval 0.63-0.67, the relative locations of these contigs along the 5DS genome zipper suggest that these contigs may be located at the junction of the deletion bins 0–0.63 and 0.63-0.67.

The physical sizes of the deletion bins along chromosome 5DS were estimated based on the contigs allocated to each bin. The size estimate was corrected by the chromosome coverage by the mapped contigs (total length of the mapped contigs/entire length of the chromosome arm), where the mapped contigs covered 54 % of the entire chromosome arm. The estimated size of the most distal deletion bin, 0.78-1.00, was a little over 49 Mb, which makes up 19.2 % of the chromosome arm, close to the cytogenetic estimate. The relatively narrow deletion bin, 0.67-0.78, was estimated to be almost 15 Mb, comprising 5.7 % of the entire chromosome arm. Strikingly, cytogenetically much smaller deletion bin, 0.63-0.67 interval was estimated as over 55 Mb, making up more than 21 % of the entire arm. These size estimates suggest that either the cytogenetic estimates of these two consecutive deletion bins are inaccurate or these bins are either under- or overrepresented in our BAC library. Finally, the most proximal deletion bin, 0–0.63, is estimated to cover 53.5 % of the chromosome arm at a size of 138 Mb. While the inconsistencies between estimates of the physical size and cytogenetic sizes may be, in part, due to the mapped loci only having 54 % coverage of the chromosome arm, it is also possible that the deletion bins are unequally represented by the genetically mapped markers, leading to enrichment of certain bins for mapped contigs.

Any bias in the number of clones in mapped contigs was not observed across deletion bins, unlike the 1BS physical map where telomeric contigs appeared to contain fewer clones than centromeric contigs on average [18], though the cumulative length of the contigs were generally smaller in the most distal bin. However, the number of clones per Mb deduced from the mapped contigs was slightly lower in the most distal deletion bin, 0.78-1.00, at 93.1 clones/Mb. These values were closer across other deletion bins, at 105.2, 105.7 and 106.6 clones/Mb for 0.67-0.78, 0.63-0.67 and 0–0.63 intervals, respectively. This suggests that although the sizes of the deletion bins vary, mapping of the contigs along these bins were generally uniform. The cumulative length of mapped contigs were 138.3 Mb, representing 53.6 % of the chromosome arm, exceeding that of 1BL physical map at 48 % [16], despite lower coverage of the chromosome arm by the overall map.

**Fig. 5:**
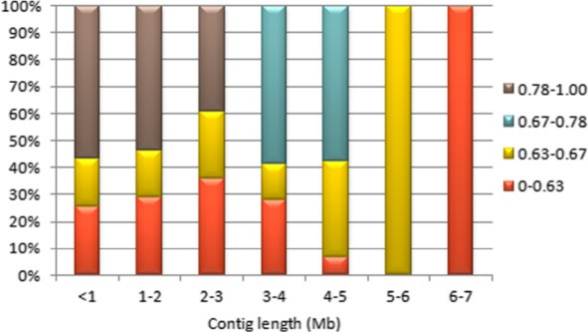
Distribution of contig lengths mapped to different deletion bins. Contigs are grouped into 1 Mb-intervals

Evaluation of the contig lengths allocated to deletion bins revealed that more than half of the contigs that were smaller than 1 Mb were mapped to the most distal deletion bin, 0.78-1.00, whereas the most proximal bin, 0–0.63, mostly contained longer contigs, where the longest contig (CTG138) was also located (Fig. 5). Along the chromosome, longer contigs tend to be located closer to centromere than telomere. Considering that the gene densities tend to increase towards the telomeric ends of the chromosomes of *Triticeae* [10], gene-associated contigs from the distal regions may have higher chances for anchoring as these regions are more likely to be covered by molecular markers. Larger contigs on proximal bins may increase the likelihood of these contigs carrying more molecular markers than shorter contigs due to their sizes.

### Small-scale genome rearrangements and syntenic perturbations

**Fig. 6:**
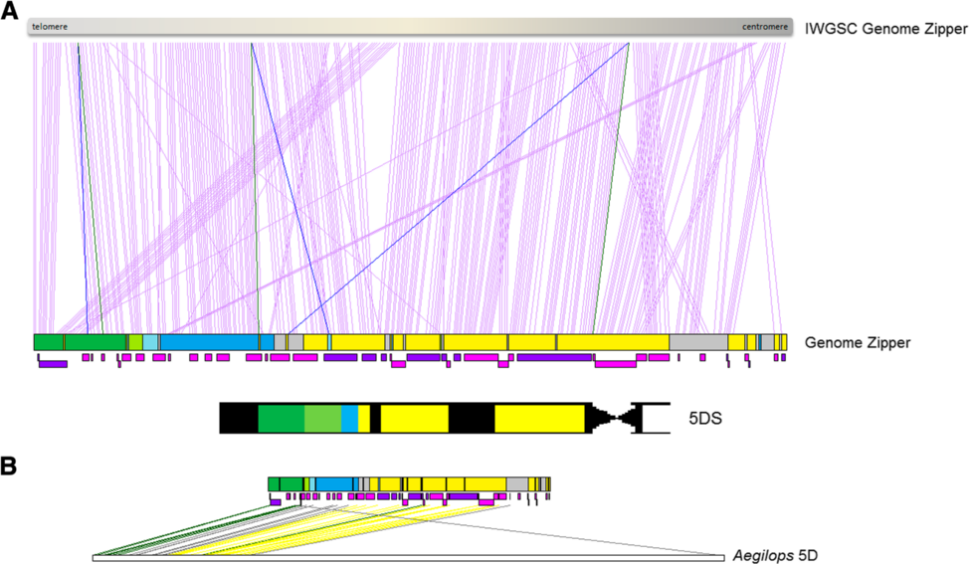
Comparisons of the genome zippers of **a** *Triticum aestivum* (Ta5DS) constructed by IWGSC and in this study, **b** Ta5DS and *Aegilops tauschii* 5D [55]. Deletion bins are color-coded where dark green, light green, blue and yellow corresponds to 0.78-1.00, 0.67-0.78, 0.63-0.67 and 0–0.63 bins, respectively. Genomic locations of the gray colored boxes are uncertain. Below Ta5DS zipper, contigs matching to *Brachypodium* orthologs are indicated as pink or purple boxes, for contigs and supercontigs, respectively. Sizes of the boxes do not necessarily reflect contig sizes, rather these indicate the number of orthologous matches for a given contig. Visualization is performed on Matlab

The relatively recent hybridization of the D-genome of *Ae. tauschii* with the tetraploid ancestral wheat genome has shaped the modern bread wheat genome, resulting in extensive homology between the ancestral and modern D-genomes [8]. The order of contigs based on the orthologous relationships with the *Ae. tauschii* genome suggested small rearrangements on the 5DS genome zipper that were not possible to deduce previously. Comparison of the ‘rearranged’ 5DS genome zipper against the genome zipper constructed by the International Wheat Genome Sequencing Consortium [6] revealed that the order of the majority of the *Brachypodium* orthologs were compatible between two zippers, suggesting that ‘genome zippers’, developed first in barley chromosome 1H [39], are powerful tools to deduce virtual gene orders when a reference sequence is not available (Fig. 6a). However, there were also striking differences between the two genome zippers, involving groups of genes. The region of orthologous genes delineated by Bradi4g00450-Bradi4g00790 and mapped to the 0.78-1.00 deletion bin in our genome zipper, was located closer to the 0–0.63 deletion bin in the genome zipper constructed by IWGSC. Intriguingly, Bradi4g05880, previously located to the 0–0.63 bin in our genome zipper, was relocated to the distal deletion bin 0.78-1.00 based on the gene order information from the *Ae. tauschii* physical map (Fig. 6a). Thus, it can be concluded that the genome zippers demonstrate the virtual gene orders to some extent but are highly dependent on the datasets used to construct them. Additionally, three *Brachypodium* orthologs (Bradi4g00980, Bradi4g02450, Bradi4g06000) appeared to be duplicated as suggested by the ordering of contigs sharing homology with these genes. These genes are indicated by blue and green lines in Fig. 6a, where the blue lines indicate putatively duplicated copies.

Although the relative ordering of 5DS contigs was largely consistent with the closely related *Ae. tauschii* 5D chromosome, comparison of the gene orders implied small rearrangements, particularly for contigs anchored and located by previously mapped EST markers (Fig. 6b). One such marker, BE443751, was originally mapped to 0.78-1.00 deletion bin; however, CTG100 anchored by this marker was eventually located on the 0–0.63 interval, which may suggest either a small-scale rearrangement after the hybridization of the D-genome or a variation among *Ae. tauschii* populations that did not exist in the ancestral D-genome (isolated green line in Fig. 6b). Moreover, CTG134 revealed homologies to two locations on *Aegilops* 5D chromosome, one of which is in the telomeric region of the long arm. This putative duplication is indicated by a gray line connecting CTG134, located on 0.78-1.00 deletion bin on the 5DS genome zipper, to a secondary location on the *Aegilops* 5D chromosome in Fig. 6b. Finally, it was not possible to differentiate borders of the deletion bins on the *Aegilops* 5D chromosome except for the most distal and proximal ones, indicated by gray lines in Fig. 6b, which may suggest additional rearrangements between the two genomes. Considering the dynamic *Triticeae* genomes, several small scale rearrangements are likely to occur through evolution [40].

**Fig. 7:**
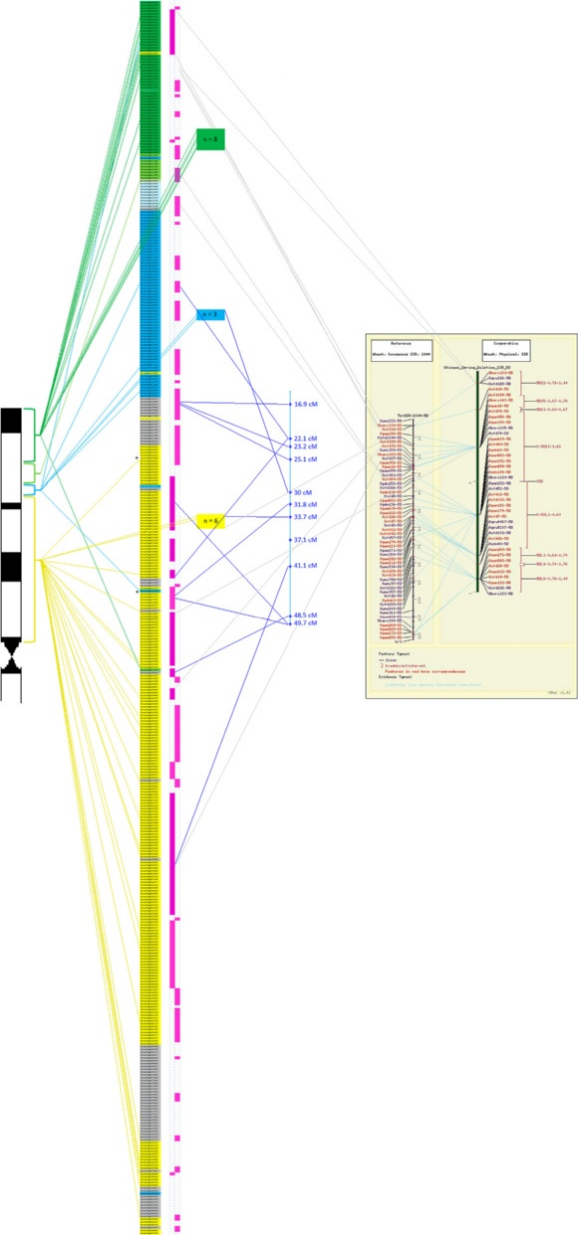
Comparison of the 5DS genome zipper integrated with the physical mapping with previously mapped markers. Bin-mapped ESTs are denoted by colored lines on the left, consensus SSR map of 5DS is given on the right (http://wheat.pw.usda.gov/GG3/maps-short) and genetically mapped COS markers are in the middle (colored in blue). Genetic distances are given in cM for COS markers. Genome zipper and contigs are depicted as in Fig. 6. Delineating syntenic genes of a putative translocation are indicated by ‘*’

The rearranged 5DS genome zipper integrated with the physical mapping data was also compared with bin-mapped ESTs and SSRs, the consensus SSR map of the 5DS chromosome (http://wheat.pw.usda.gov/GG3/maps-short) and genetically mapped COS markers (Fig. 7). The previously bin-mapped ESTs and SSRs were generally consistent with our map, except for a few EST markers matching syntenic genes or contigs that were positioned in a different deletion bin, suggesting small-scale rearrangements. Curiously, the region of the chromosome arm delineated by Bradi4g02900-Bradi4g03750 (Fig. 7, indicated with ‘*’) was marked by ESTs mapped to 0–0.63 and 0.63-0.67 bins, respectively, which may point to an intrachromosomal inversion. The few SSR markers from the consensus map exhibiting inconsistencies with the ordering of syntenic genes along 5DS may also indicate small-scale rearrangements, while it is also possible that the consensus map may not reflect SSR orders on this chromosome arm accurately. The inconsistencies between the cytogenetic positions of the mapped COS markers and the syntenic genes/contigs to which they correspond to is intriguing; it can be speculated that while these markers are derived from conserved genes, the order of the genes between the wheat and rice genomes from which these markers were designed [41] may not be well-conserved.

### Gene space of chromosome 5DS assessed by conserved probe hybridizations

Microarray hybridization of the conserved probes, derived from reciprocal matches between 1.34x survey sequences and three annotated grass genomes, enabled the exploration of the gene space of 5DS. Of these conserved probes, 1,306 were assigned to contigs of the 5DS physical map. These probes corresponded to 95, 41, 105 and 231 conserved genes in deletion bins 0.78-1.00, 0.67-0.78, 0.63-0.67 and 0–0.63, respectively. Considering the cumulative lengths of the contigs assigned to each deletion bin, these figures suggested gene densities that vary between 3.17-5.17 genes/Mb along the chromosome arm (Table 3). Intriguingly, the relatively narrow deletion bin, 0.67-0.78, exhibited the highest density of genes at 5.17 genes/Mb. A similar gene density of the 5.1 genes/Mb was reported for the chromosome arm 1AS towards the telomeric end [17]. Gene densities calculated by the cumulative length of contigs assigned to each bin supported our estimates of the physical sizes of the deletion bins, particularly for 0.63-0.67 interval, which contrasted dramatically from the cytogenetic estimate. In fact, if the cumulative length of all mapped contigs were distributed according to the cytogenetic estimates, 0.63-0.67 deletion bin would have a gene density of 19 genes/Mb, which would be highly unlikely. The comparable gene density for the 0.67-0.78 deletion represented by only two contigs in our final map further corroborates our estimates of the bin sizes and confirms that our data is adequately representative of the chromosome arm.

**Table 3:** Gene content and organization of 5DS assessed by mapped contigs, distributed along deletion bin intervals

	Syntenic		Non-syntenic		Total		In islands		Isolated		
Interval	Number	Density	Number	Density	Number	Density	Number	Density	Number	Density	Cumulative length (Mb)
0-0.63	131	1.80	100	1.37	231	3.17	192	2.63	39	0.53	72.92
0.63-0.67	44	1.48	61	2.05	105	3.53	83	2.79	22	0.74	29.71
0.67-0.78	13	1.64	28	3.53	41	5.17	33	4.16	8	1.01	7.93
0.78-1.00	20	0.75	75	2.83	95	3.58	79	2.98	16	0.60	26.54

**Fig. 8:**
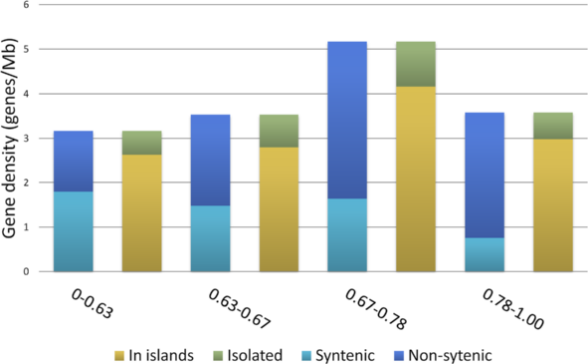
Gene space content and organization of 5DS assessed by conserved probes hybridizing to MTP clones

The gradient of the syntenic gene density along deletion bins was not correlated with the overall gradient of gene density (Pearson’s correlation coefficient r = 0.16, p-value = 0.84), while the gradient of non-syntenic gene density was correlated (r = 0.87, p-value = 0.13) though the correlation was not highly significant, which may be due to the unusual gene density of the 0.63-0.67 deletion bin. Assuming that genes located on the same or overlapping BAC clones constitute “islands” of genes [16, 18], organization of the gene space along 5DS also demonstrated dominance of gene islands over isolated genes along each deletion bin (Fig. 8, Table 3), consistent with previous findings [10, 16, 18]. While the gradient of overall gene density did not correlate significantly with the syntenic and non-syntenic genes, the density of the genes found in islands was highly correlated with the overall gradient (r = 0.9956, p-value = 0.0044) which was not quite the case with isolated genes (r = 0.9509, p-value = 0.0491).

**Fig. 9:**
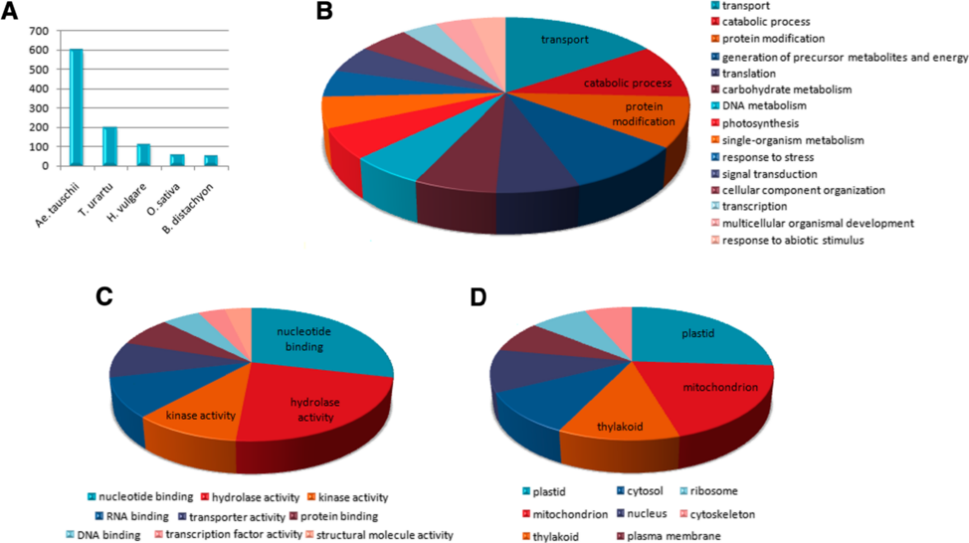
Blast2GO statistics and annotations for the syntenic read probes. **a** Top BLAST-hit species, *x-axis* species name, *y-axis* number of BLAST hits. **b**, **c**, **d** Blast2GO annotations for Biological Process, Molecular Function and Cellular Component, respectively

To further explore the 5DS gene space, conserved read probes hybridizing to MTP clones were functionally assessed. Of the total 6,996 such probes, 1,306 giving positive signals under high stringency (see Materials and Methods) were annotated using the Blast2GO tool [42]. The remaining probes may have been eliminated due to the stringency measures applied to hybridization results or may have been located on singleton clones which were not included in the microarray experiments. Functional annotations of these conserved read probes gave insights into the gene space of 5DS (Fig. 9). Interestingly, the sequence comparison of the positive conserved probes to non-redundant *Viridiplantae* proteins yielded the most BLAST hits in *Aegilops tauschii* followed by *Triticum urartu* (Fig. 9a). This implies that, on the basis of the conserved microarray probes hybridizing to 5DS MTP clones, the 5DS chromosome arm shares extensive similarity with the D genome of *Ae. tauschii*. The relatively recent hybridization of *Ae. tauschii* with the AABB progenitor, giving rise to the hexaploid genome of *Triticum aestivum*, has allowed only restricted inter-chromosomal recombination compared to the A and B-genomes. This may explain why the majority of the syntenic probes on 5DS map yielded extensive homology to no species other than its ancestor, *Ae. tauschii*. There were also significant matches with the completely annotated proteomes of *Oryza sativa* and *Brachypodium distachyon* that emphasize the close evolutionary relationships among grasses. The GO terms assigned for Biological Process (BP), Molecular Function (MF) and Cellular Component (CC) terms revealed a range of processes, functions and locations mostly consistent with the annotations obtained from survey sequences generated from the whole of chromosome 5D [25]. BP terms for probes in conserved genes were enriched for transport, catabolic process and protein modification, among others (Fig. 9b). The 5DS gene space is likely to contribute to several processes to similar extents, rather than specializing on one or a few processes. In the case of MF terms, however, three functions appeared to dominate others. Nucleotide binding, hydrolase activity and kinase activity, together accounted for over 60 % of all MF terms (Fig. 9c). Intriguingly, hydrolase activity was also prominent in the secretome of an apple pathogen *Venturia inaequalis* closely related to the wheat pathogen *Pyrenophora tritici-repentis* [43] and was also central to the transcriptome of the wheat pest *Heterodera avenae* [44]. While the prominence of the hydrolase activity term among the probes may be reflected in the ‘catabolic process’ BP term (Fig. 9b), which accounts for a considerable portion of all BP term annotations, it could also be related to defense mechanisms, though in a general sense rather than specialized stress-response activities. From this perspective, genes involved in hydrolysis-related processes may be enriched on 5DS chromosome arm. CC terms revealed a higher than expected contribution of sequences to be related to the plastid or mitochondrion, which is also intriguing (Fig. 9d). As these organelles possess their own genetic material, it is tempting to speculate that 5DS was involved in the transfer of genes from the organellar DNA to the nucleus [45]. Considering that photosynthesis or energy-related process did not dominate BP terms, the large contribution of these organelles to CC terms is curious. Overall, while BP terms did not point to a specific process to which 5DS contributes to, the weighting of MF and CC terms may suggest that this chromosome arm includes families of genes devoted to carry out similar and specific functions, which would be consistent with the transcriptional autonomy of sub-genomes without the genome-wide dominance of one sub-genome over others [6].

Technological advances in sequencing technologies have paved the way for exploration and exploitation of crop genomes through genome sequencing. Though it has been more than a decade since the first plant genome sequence was published for *Arabidopsis thaliana* [46], crop genome sequencing has been hindered by major challenges such as large genome size and complexity. Nonetheless, ongoing efforts have culminated into the very recently published draft genome sequence of wheat and the reference sequence of its largest chromosome, which was built on a BAC-based physical map [6, 14]. Noticeably, BAC-based physical maps will be an integral part of the reference sequencing of the entire wheat genome, through providing a framework for the sequence assembly. Accordingly, the high quality physical map of 5DS presented in this study, will not only contribute to the identification of genes through map-based cloning and but also aid in the assembly of the reference sequence of this chromosome.

## Conclusions

Due to its allohexaploid nature and high repetitive content, sequencing of the entire bread wheat genome is a daunting task. Ongoing efforts towards producing a whole genome sequence have focused on the construction of physical maps of individual chromosomes which will guide assembly of the sequences. Accordingly, the high quality physical map of 5DS presented in this study, with a density of 10.5 markers/Mb, will not only contribute to the identification of genes through map-based cloning and but also aid in the assembly of the reference sequence of this chromosome. The insights gained into the 5DS chromosome arm suggested an unusual gene density for one of the deletion bins, implying an inaccuracy in the cytogenetic size estimate of this bin. Additionally, the improved virtual gene order revealed gene duplications and small rearrangements. Consequently, this study provides new insight into the genome structure and organization of the 5DS chromosome arm, which will be crucial for understanding the molecular biology of this chromosome and for its future sequencing to reference quality.

## Methods

### BAC library construction, fingerprinting and map assembly

The short arm of wheat chromosome 5D (5DS) was sorted by flow cytometry from a double ditelosomic line (2n = 40 + 2t5DS + 2t5DL) of *Triticum aestivum* L. cv. Chinese Spring provided by Prof. B.S. Gill (Kansas State University, Manhattan, USA). Preparation of aqueous suspensions of intact mitotic chromosomes from synchronized root tips of young seedlings, and chromosome sorting was done as described by Vrána *et al.* [11]; the purity in flow-sorted fractions was determined by fluorescence in situ hybridization (FISH) with probes for *Afa* and telomeric repeats [47]. Flow-sorted chromosomes were embedded in agarose miniplugs and used to construct 5DS-specific BAC library as described by Šimková *et al.* [48]. Of the 5DS BAC library, 26,112 BAC clones, with an average insert size 143 kb and giving 12.5x coverage of the chromosome arm, were fingerprinted using SNaPshot™ High-Information Content Fingerprinting (HICF) procedure [35]. The fingerprints were then processed to remove bands stemming from either the vector or the host gDNA, bands resulting from partial digestion or star activity, unreliable bands of unexpected sizes and background noise using the FingerPrint Background removal (FPB) software [49]. True bands in the range of 50–500 bp were analyzed by GenoProfiler software [50] to eliminate cross-contaminations and negative controls.

### Preliminary map construction

A total of 21,656 good-quality fingerprints were used to construct the preliminary physical map of 5DS. Initially, two software programs, FingerPrintedContig (FPC) [31] and Linear Topology Contig (LTC) [32], were separately used to construct physical map of 5DS. For both preliminary assemblies, previously established and optimized parameters for FPC [13, 15] and LTC [15, 32] were used, which are summarized below.

For the FPC assembly, the previously described methodology was followed [13, 15]. Briefly, the initial assembly of the BAC clones was performed at a high stringency with a Sulston Score probability cut-off of 1e^−75^ which was then incrementally increased at six steps to 1e^−45^, adding singleton clones to the ends of the existing high-confidence contigs and merging contig ends at each step. Contigs with 6 or less clones were excluded. The final FPC assembly consisted of 350 contigs with an N50 of 1,141 kb (Table 1).

LTC assembly was also carried out as previously described [15]. In brief, the initial net of significant clone overlaps was established at a less stringent Sulston Score probability cut-off of 10^−15^. Q-clones and Q-overlaps were removed at cut-offs of 10^−15^ and 10^−25^, respectively. The first round of adaptive clustering was conducted at the cut-off of 10^−15^, and the cut-off was reduced to 10^−33^, thereby increasing the stringency, in 6 steps, to split non-linear contigs. Persistent non-linear contigs were visualized and inspected individually to identify clone sources causing branching in non-linear contigs. Thirteen such clones were detected and excluded from the next round of adaptive clustering. The second round of adaptive clustering yielded 164 contigs, including 44 short contigs of sized <6 clones (Additional File 2).

### Minimum tiling path and BAC pooling

The minimum tiling path (MTP) was generated separately by FPC and LTC software from the respective assemblies. Upon the observation that the MTP selected by the LTC software included several buried clones, an alternative MTP of 2155 clones was selected via FPC with the parameters described previously [15], and were then tested by the LTC to confirm the clone overlaps. As the LTC program builds initial net of clone overlaps at a Sulston Score probability cut-off of 10^−15^, any overlaps that are only significant at cut-offs above 10^−15^ are considered unreliable. Within a contig, if the clone orders are correct but the clone overlaps are unreliable, the contig is deemed reliable, however, gaps may remain at the sequence level. In order to present an adequate source for reference sequencing and avoid such gaps at the sequence level, clone overlaps marked as ‘unreliable’ by LTC is reinforced with additional clones, as follows: For overlaps that were significant only at a cut-off of 10^−14^ or above, 210 additional clones covering the same overlap were picked manually. Lastly, 163 clones that were considered as Questionable-clones (Q-clones) and were excluded from the physical map were added to the MTP, in order to assess these clones for possible use as bridge clones in the final assembly. Overall, 2,528 BAC clones were included in the MTP.

The 2,528 MTP clones from the original 5DS BAC library were re-arrayed into 7 384-well plates. Through a 3-dimensional (3D) pooling strategy previously described [13], each clone was grown individually and then pooled into 16 row, 24 column and 7 plate pools in 96-well plates, to reduce the number of samples to be screened.

### Marker design, selection and MTP pool screening

In order to anchor contigs of the 5DS physical map with molecular markers, 1.34x coverage 454 Roche reads originating from 5DS [25] were utilized to design Insertion Site-Based Polymorphism (ISBP) and Simple Sequence Repeat (SSR) markers using IsbpFinder.pl and IsbpSort.pl scripts and SciRoKo program, respectively [51, 52] (Additional file 3). A total of 16,727 high-confidence ISBP markers, which contain a unique non-repetitive sequence flanking the junction site of a repetitive element, were defined, of which 99 high-confidence ISBP markers were tested on MTP pools. Additionally, MTP pools were screened by genetically mapped molecular markers to integrate 5DS physical map with the available genetic maps. For this purpose, 2 gene based markers (Pina-D1, Pinb-D1), 23 Simple Sequence Repeat (SSR) markers and 63 Expressed Sequence Tag (EST) markers were retrieved from GrainGenes database 2.0 (http://wheat.pw.usda.gov). A total of 13 published Conserved Orthologous Set (COS) markers were also included in MTP screening [41].

Screening of MTP pools was performed in a 10 μl PCR reaction volume, using Taq polymerase (Fermentas) as follows: 1 μl 10X KCl Buffer (−MgCl_2_), 0.8 μl 25 mM MgCl_2_, 0.2 μl 2.5 mM each dNTP, 0.25 μl 10 μM Forward Primer, 0.25 μl 10 μM Reverse Primer, 1 μl Template DNA, 0.05 μl Taq Polymerase, 6.45 μl dH_2_O. Reaction conditions were as follows: Initial denaturation, 94 °C 5 min, 35 cycles of {Denaturation, 94 °C 30s, Annealing, variable 30s, Extension 72 °C 30s}, Final extension 72 °C 7 min. For markers giving multiple hits in row, column and/or plate pools, colony PCRs were performed using the original MTP clones to determine which clones they were derived from.

### Microarray design and hybridization

An Agilent SurePrint G3 Gene Expression Custom Microarray, 8x60k format (Agilent Technologies) was designed using three sources of sequences as probes: 1) Genetically mapped gene/marker sequences, 2) Conserved 5DS sequence reads [25], 3) ISBP markers designed from 5DS survey sequences [25]. For Gene/Marker probes, 7 genes mapped on 5DS (*Pina-D1*, *Pinb-D1*, *Gsp-1*, *MdH-D3*, *Nor-D3*, *Pro2*, *5S-RNA-D2*), 13 COS markers, 122 EST markers and 20 SSR markers (a total of 162 gene/marker sequences) were used to design probes with these parameters: probe length = 60 bp, probes per target = 5, preferred probe Tm 85 °C with a Tm matching methodology. Additionally, 3 Single Nucleotide Polymorphisms (SNPs) mapped to 5DS [53] were used to design probes according to the above criteria. Lastly, 109 SNPs mapped to 5D by Illumina sequencing [54] were included as probes and due to the short lengths of these sequence reads, each sequence was included 5 times in the overall design. For conserved read probes, 1.34x coverage 454 Roche sequence reads derived from 5DS were blasted against related grass proteomes of *Brachypodium distachyon*, *Oryza sativa* and *Sorghum bicolor*. Reciprocal best hits in blastx and tblastn searches filtered against a minimum alignment length of 30 amino acids and 75 % similarity at an e-value of 10^−6^ or lower, were retained as ‘conserved’ read sequences [25]. 6,996 such sequences were used to design probes, using the same parameters as above. For the remaining features on the array, 5,120 ISBP marker sequences (amplicon size >150 bp) designed from 5DS survey sequences were included as probes with the same parameters. The final design was comprised of 1,370 probes for genetically mapped genes/markers, 34,980 probes derived from conserved gene reads, and 25,600 probes for ISBP markers designed from 5DS survey sequences.

For the microarray hybridization, MTP pools were labeled with Cy3 and Cy5 using the SureTag DNA Labeling Kit (Agilent Technologies, Cat. No. 5190–3400) following the manufacturer’s instructions. Labeled pools were hybridized to the probes in pairs in a dye-swap design. Hybridization and wash steps were performed as indicated by the manufacturer, and the arrays were scanned with NimbleGen MS 200 microarray scanner (Roche NimbleGen, Inc.) at 2 nm resolution with autogain. Agilent Feature Extraction Software (v. 11.5.1.1) was used to extract fluorescence data from the scanned images. Data normalization and deconvolution was performed using custom R scripts independently for row, column and plate pools, as previously described [15, 36]. Three different C values for set for each pool type, such that 2.8, 1.6 and 2.6 for column, plate and row pools, respectively, and these were compared to Student’s *t*-Test results at p-value < 0.01. Additionally, two sets of less stringent C values (medium confidence: 2.6, 1.6, 2.4, and, low confidence: 2.4, 1.4, 2.2 for column, plate and row pools, respectively) were used to anchor additional contigs at the same p-value. For probes with multiple positive pools passing both tests, only those that are found on overlapping BAC clones of the preliminary map were retained.

### Contig elongation and contig ordering

Using the ‘Elongation’ feature of the LTC program reported recently [18], contig ends from the preliminary map were manually tested for possible overlaps with other contigs at a less stringent cutoff of 10^−15^. In order to locate these contigs and supercontigs into the deletion bins along 5DS, molecular markers assigned to MTP clones through PCR or microarray hybridization were used. Since ISBP markers designed from the 1.34x coverage 5DS survey sequence reads lacked a genetic location, those ISBP markers that were mapped by PCR were also screened on homozygous deletion lines of 5DS-2 and 5DS-5, which are 0.78 and 0.67 of the full length chromosome arm, respectively. The contigs within each deletion bin were ordered based on the order of orthologous sequences on the recently published physical map of the *Aegilops tauschii* genome [55]. Orthologous sequences were determined based on similarity searches against positive probe sequences at an e-value of 10^−10^. For contigs lacking an *Ae. tauschii* ortholog, *Brachypodium* orthologs and their ordering along the 5DS genome zipper constructed by our group was used.

### Functional annotations of conserved microarray probes

Conserved read probes passing the statistical tests for microarray hybridization were annotated using Blast2GO tool [42]. Initially, probe sequences were blasted against a local non-redundant *Viridiplantae* protein database using BLAST+ command line applications, v.2.2.27 [56] at an e-value of 10^−6^. All blast hits were then mapped and annotated using the Blast2GO application with default settings for plants. Finally, multi-level charts were generated for Biological Process, Molecular Function and Cellular Component terms, individually.

## Additional files

Additional file 1:
**Histogram of relative fluorescence (flow karyotype) obtained after flow cytometric analysis of DAPI-stained mitotic chromosomes of double ditelosomic line 5D of** ***Triticum aestivum*** **cv. Chinese Spring.** The flow karyotype consists of three composite peaks I – III representing groups of wheat chromosomes, peak of chromosome 3B, and clearly discriminated peaks of telocentric chromosomes 5DS and 5DL. Inset: Images of flow-sorted 5DS after FISH with probes for Afa repeat (green) and telomeric repeat (red). The chromosomes were counterstained by DAPI (blue). X-axis: Relative fluorescence intensity. Y-axis: Number of particles. Additional file 2:
**The preliminary map details of 5DS constructed by LTC.**
Additional file 3:
**Newly designed ISBP and SSR markers for 5DS.**
Additional file 4:
**Molecular markers assigned to clones via PCR amplification or microarray hybridization.**
Additional file 5:
**Details of the supercontigs with connecting clones.**
Additional file 6:
**Deletion bin map of 5DS.** Markers in black are physically anchored; markers in blue are putatively assigned via microarray. Markers anchored by both approaches are indicated in green.

## Abbreviations

BAC: Bacterial Artificial Chromosome
BARC: USDA-ARS Beltsville Agricultural Research Center
BES: BAC-end sequences
COS: Conserved Orthologous Set
DAPI: 4',6-diamidino-2-phenylindole
EST: Expressed Sequence Tag
FISH: Fluorescence in situ Hybridization
FPC: FingerPrintedContig
GWM: Gatersleben Wheat Microsatellite
HICF: High-Information Content Fingerprinting
ISBP: Insertion Site-Based Polymorphism
IWGSC: International Wheat Genome Sequencing Consortium
LTC: Linear Topological Contig
MTP: Minimum Tiling Path
PCR: Polymerase Chain Reaction
Q-clones: Questionable clones
SNP: Single Nucleotide Polymorphism
SSR: Simple Sequence Repeat
WMC: Wheat Microsatellite Consortium
