# Supplementary figures and images for: The physical map of wheat chromosome 5DS revealed gene duplications and small rearrangements
Source: BMC Genomics. 2015 Jun 13;16(1):453. doi: 10.1186/s12864-015-1641-y (PMC4465308; doi:10.1186/s12864-015-1641-y)

## Slide 1
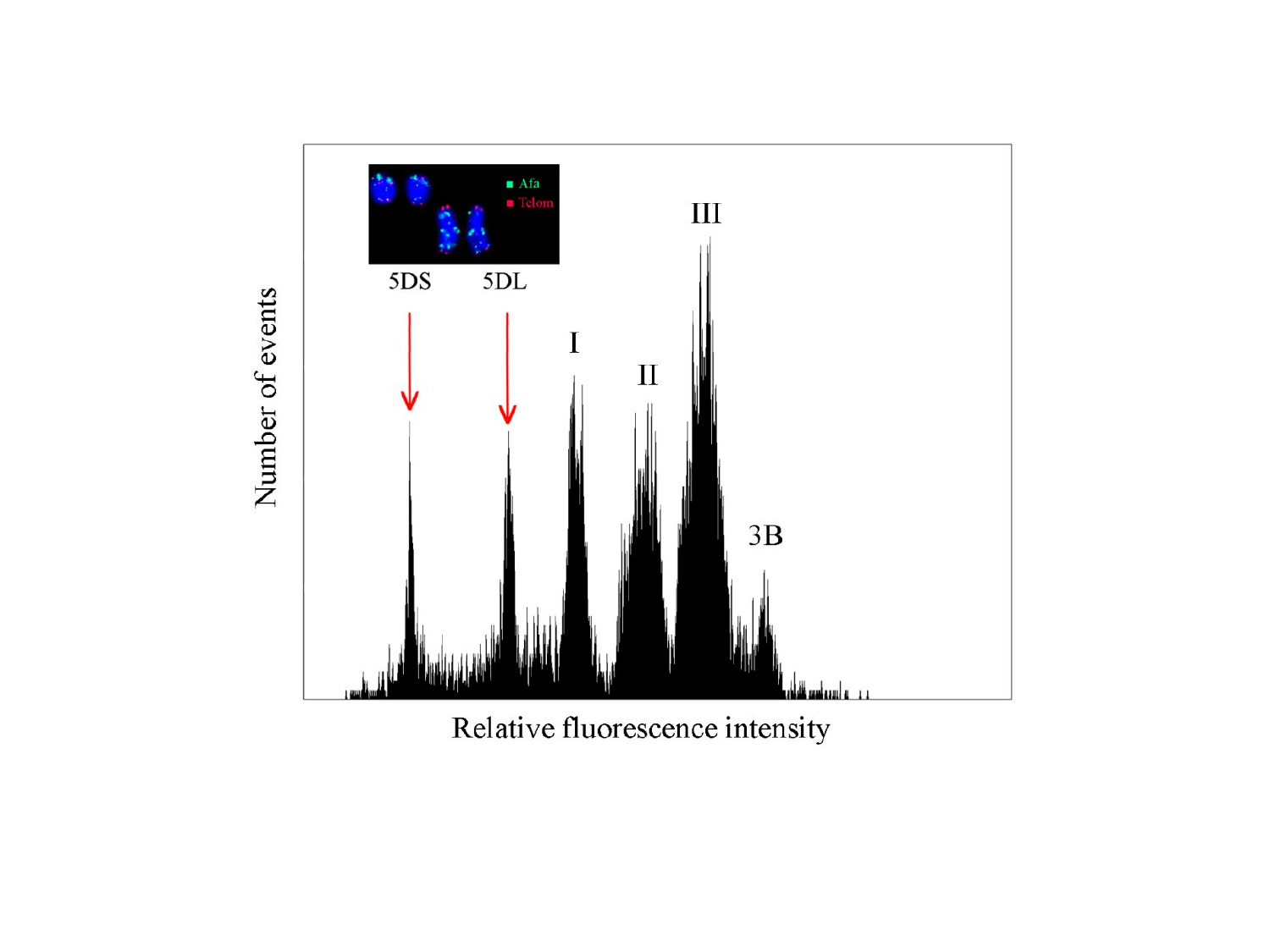

Supplement: Additional file 1: — Histogram of relative fluorescence (flow karyotype) obtained after flow cytometric analysis of DAPI-stained mitotic chromosomes of double ditelosomic line 5D of Triticum aestivum cv. Chinese Spring. The flow karyotype consists of three composite peaks I – III representing groups of wheat chromosomes, peak of chromosome 3B, and clearly discriminated peaks of telocentric chromosomes 5DS and 5DL. Inset: Images of flow-sorted 5DS after FISH with probes for Afa repeat (green) and telomeric repeat (red). The chromosomes were counterstained by DAPI (blue). X-axis: Relative fluorescence intensity. Y-axis: Number of particles. [file 12864_2015_1641_MOESM1_ESM.pptx]
